# Majority of *T. gondii* seropositive chickens (*Gallus domesticus*) in Central Ethiopia carries the infective parasite

**DOI:** 10.1186/s13028-014-0060-4

**Published:** 2014-09-24

**Authors:** Endrias Zewdu Gebremedhin, Gebregergs Tesfamaryam, Reta Duguma, Getachew Tilahun, Vincenzo Di Marco, Maria Vitale

**Affiliations:** Department of Veterinary Laboratory Technology, Ambo University, Faculty of Agriculture and Veterinary Sciences, P.O.Box 19, Ambo, Ethiopia; Jigjiga University, College of Veterinary Medicine, P.O.Box 307, Jigjiga, Ethiopia; Department of Clinical Studies, Addis Ababa University, College of Veterinary Medicine and Agriculture, P.O.Box 34, Debre Zeit, Ethiopia; Addis Ababa University, Aklilu Lemma Institute of Pathobiology, P.O.Box 1176, Addis Ababa, Ethiopia; Italian National Reference Centre for Toxoplasmosis at Istituto Zooprofilattico Sperimentale della Sicilia A. Mirri, Palermo, Italy

**Keywords:** Free range chicken, DAT, bioassay, *Toxoplasma gondii*, Central Ethiopia

## Abstract

**Background:**

The prevalence of *Toxoplasma gondii* in free range chickens is a good indicator of the prevalence of *T. gondii* oocysts in the environment. The aim of this study was to isolate *T. gondii* parasites from heart and brain of seropositive free range (FR) chickens.

**Findings:**

Isolation of *T. gondii* from pooled heart and brain of 41 direct agglutination test (DAT) positive (≥1:40) free range chickens (*Gallus domesticus*) was carried out by bioassay in mice. *T. gondii* specific antibodies in mice were assayed by DAT and microscopy was employed for detection and enumeration of brain tissue cysts. Overall, bioassay was positive in 29 (70.7%) chicken samples. *T. gondii* tissue cysts were isolated from 59% (24/41) of bioassayed chickens: from 2 of 7 chickens with a titer of 1: ≤ 60, 2 of 5 with titer 1: 180, 6 of 8 with titer 1: 540, 10 of 15 with titer 1: 1620, 1 of 2 with titer 1: 6000, 2 of 3 with titer 1:18000, 1 of 1 with titer 1:54000. None of the isolates was pathogenic for mice. Tissue cysts were detected from 61% of seropositive mice (DAT ≥ 1:40). Generally, tissue cyst counts per brain of mouse were low (mean: 132.7 ± 84.4; range: 47–352).

**Conclusions:**

Majority of *T. gondii* seropositive chickens (*Gallus domesticus*) in Central. Ethiopia carries the infective parasite. Tissues from the free range chicken might be a source infection for animals and humans.

## Findings

*Toxoplasma gondii* is a zoonotic, obligate intracellular protozoan parasite that has the capacity to infect all warm blooded animals. Toxoplasmosis is usually subclinical; however the disease is an important cause of congenital problems and abortion in sheep, goats and women [1,2].

Bioassay in mice or cats has been used as a highly sensitive and specific test to isolate viable *T. gondii*. Recently, as an alternative to bioassay Opsteegh *et al*. [3] developed detection method using magnetic capture of *T. gondii* DNA followed by quantification of the parasite using quantitative real-time PCR.

So far two published information are available about isolation of viable *T. gondii* from any host in Ethiopia [4,5]. The present study was undertaken from September, 2012 to May, 2013 with the aim of isolation of *T. gondii* from heart and brain of seropositive free range (FR) chickens in Ambo, Adea and Fentale districts of Oromia Regional State, Central Ethiopia. Ambo district (37° 32’ to 38° 3’ E and 8° 47’ to 9° 20’ N) is found in West Shewa Zone while Adea (38° 58’ E to 39° 22’ E and 08° 22’ N to 8° 56’ N) and Fentale (39.93° E to 39° 56’0” E and 8.975° N to 8.58’30” N) districts are located in East Shewa Zone of Oromia Region, Central Ethiopia.

Forty-one DAT positive backyard chickens out of 183 seropositive chickens identified during seroepidemiological study [6] were randomly sampled depending on willingness of owners to sell for the present study. The chickens were killed by cervical dislocation. Bioassay was performed as described by Dubey [2]. Briefly, the brain and heart of each chicken were pooled, homogenized, digested by incubating in acidic pepsin, filtered, centrifuged and the sediment neutralized with sodium bicarbonate. After another centrifugation the pellet was resuspended in saline mixed with penicillin (1000 U/ml) and streptomycin (100 μg/ml) and the suspension was inoculated intraperitoneally into Swiss white albino mice (National Veterinary Institute, Debre Zeit, Ethiopia). Five mice per chicken sample, a total of 205 mice, were used for the study. Five non-infected mice were kept separately as negative controls.

On day 45, surviving mice were bled during terminal anesthesia with di-ethyl ether (Biolab laboratories ltd, Israel). Blood samples were allowed to clot; centrifuged and sera were collected in cryovials. Sera samples were examined for the presence of antibodies (IgG) against *T. gondii* by the direct agglutination test (Toxoscreen DA, biomerieux®, France) following the protocol of the manufacturer. Sera were assayed at a screening dilution of 1:40 and 1:4000. A positive result at 1:40 or 1:4000 or both was considered indicative of *T. gondii* exposure.

Brains of all mice were examined for tissue cysts as described by Dubey [2]. Brains of all mice were removed by sagittal dissection, homogenized in 1 ml phosphate buffer saline (PBS) (pH = 7.2) by using a mortar and pestle and then examined for tissue cysts. The numbers of cysts in three aliquots of each 10 μl were counted under microscope with a 10X objective lens, summed and converted to a count per mouse brain [7]. A bioassay was considered positive if at least one *T. gondii* cyst was detected in any of the five inoculated mice or any of the mouse serum or mice sera reacted positively for DAT.

STATA version 11.0 for Windows (Stata Corp. College Station, USA) was used to analyze the data. Descriptive statistics were used to summarize the data. Association of cyst positivity (dependent variable) with independent variables (altitude, district, breed, sex, age, residence, and DAT titer of chicken) was assessed using Chi-square test and logistic regression. Non-collinear variables that presented a *P*-value of < 0 · 25 in univariable analysis were included in the multivariable logistic regression model. Results were considered significant at *P* ≤ 0.05.

This research project was approved by the animal ethical committee of the College of Veterinary Medicine and Agriculture, Addis Ababa University.

Overall, bioassay was positive in 29 (70.7%) chicken samples (22 samples were positive for both the tissue cysts and *T. gondii* specific antibodies; 5 samples were positive for serology alone and 2 samples were positive for cyst alone (Table 1). *T. gondii* was isolated from 24 (59%) of 41 seropositive chicken (≥1:40) hearts and brains bioassayed in mice. Serological test (DAT) of survived mice (n = 204) revealed seropositive result in 100 (49%) of the mice used for isolation, whereas tissue cysts were detected from 61 (61%) of seropositive mice. Of the 204 mice brain examined microscopically, 61 (30%) were positive for *T. gondii* tissue cysts (Table 1).

**Table 1:** **Results of bioassay positive chicken samples** (**n** = **5 mice**/**chicken sample**)

**District**	**Id**	**Chicken**			**DAT titer of seropositive chicken**	**Bioassay in mice**
		**Breed**	**Sex**	**Age in months**		**Seropositive mice,** **cyst positive mice**
Ambo	Ch159	Local	F	12	180	4, 0
	Ch55	Local	F	12	1620	3, 1
	Ch52	Local	F	12	18000	5, 1
	Ch140	Local	F	12	1620	1, 1
	Ch40	Local	F	12	1620	4, 5
	Ch142	Local	F	12	1620	5, 4
	Ch42	Local	F	9	≤60	5, 5
	Ch192	Local	F	11	1620	0, 1
	Ch191	Local	F	12	54000	5, 5
	Ch54	Local	M	8	≤60	5, 2
	Ch26	Local	F	8	540	5, 4
	Ch28	Local	F	7	6000	5, 0
	Ch38	Exotic	F	12	1620	4, 3
	Ch31	Local	F	8	540	5, 5
	Ch27	Local	F	9	18000	5, 5
	Ch35	Local	F	6	1620	3, 0
Adea	Ch310	Local	M	24	540	0, 1
	Ch392	Local	F	13	540	5, 1
	Ch414	Local	F	12	540	5, 3
	Ch397	Local	F	9	1620	4, 0
	Ch404	Local	F	10	540	5, 3
	Ch411	Local	F	13	1620	4, 1
	Ch396	Local	M	8	180	5, 4
	Ch331	Local	F	9	≤60	3, 0
	Ch348	Local	M	4	1620	4, 1
	Ch330	Local	F	9	180	1, 1
	Ch350	Local	F	4	1620	1, 1
Fentale	Ch548	Local	M	12	1620	4, 2
	Ch569	Local	F	9	540	1, 1

The table shows results of those bioassay positive samples (29) out of 41 seropositive chicken samples bioassayed. Sixteen chickens from Ambo, 11 from Adea and 2 from Fentale districts were bioassay positive. *m.e* = mice examined.

All inoculated mice survived the infection and manifested no clinical signs except 1 mouse which died 2 days post-inoculation perhaps due to infection or error during inoculation. All inoculated mice were examined microscopically for *T. gondii* regardless of DAT result. *T. gondii* parasites were isolated from 2 of 7 chicken with titer of 1: ≤ 60, 2 of 5 with titer 1: 180, 6 of 8 with titer 1: 540, 10 of 15 with titer 1:1620, 1 of 2 with titer1: 6000, 2 of 3 with titer 1:18000, 1 of 1 with titer 1:54000 (Table 1). Among the independent variables investigated at chicken level for association with cyst isolation rate, only DAT end titer of chicken was found to be significantly associated (*P* < 0.05) (Table 2).

**Table 2:** **Association of cyst positivity in mice after bioassay of chicken tissues with potential explanatory variables**

**Variable and category**	**No. examined**	**No. cyst positive (%)**	**95%** **confidence interval**
District			
Fentale	6	2 (33.3)	4.3 – 77.9
Adea	15	9 (60.0)	32.3 – 83.7
Ambo	20	13 (65.0)	40.8 – 84.6
Altitude			
Low land	6	2 (33.3)	4.3 – 77.9
Mid land	25	16 (64.0)	42.5 – 82.0
High land	10	6 (60.0)	26.2 – 87.8
Breed			
Local	40	23 (57.5)	40.9 – 73.0
Exotic	1	1 (100.0)	0.025 – 1**
Sex			
Male	8	5 (62.5)	24.5 – 91.5
Female	33	19 (57.6)	39.2 – 74.5
Age			
≤ 6 months	7	2 (28.6)	3.7 – 71.0
7- 12 months	10	5 (50.0)	18.7 – 81.3
≥ 13 months	24	17 (70.8)	48.9 – 87.4
Residence			
Rural	22	10 (45.5)	24.4 – 67.8
Urban & periurban	19	14 (73.7)	48.8 – 90.9
DAT end titer			
≤ 60	8	2 (25.0)	3.2 – 65.1
180	5	2 (40.0)	5.3 – 85.3
540	7	7 (100.0)	59.0 – 1**
1620	15	10 (66.7)	38.4 – 88.2
6000	2	0 (0.0)	0 – 84.2 **
18000	3	2 (66.7)	9.4 – 99.2
54000	1	1 (100.0)	0.025 – 1**

*DAT end titer of chickens was significantly associated with isolation of brain tissue cysts in mice (*P* = 0.037). The likelihood of cyst positivity is higher from chicken with high antibody titer, **one–sided 97.5% confidence interval.

Among 61 cyst positive mice, 57 and 4 were DAT positive and negative, respectively. Among the cyst positive mice, the mean ± standard deviation [SD] of cyst count per mice brain was 132.7 ± 84.4 (range: 47–353). The mean ± SD of cyst count per mice brain was 123.4 ± 89.9 for Adea district while it was 128.7 ± 74.5 and 136.6 ± 84.5 for Fentale and Ambo districts, respectively. There was no significant difference in mean cyst count between districts.

Results of multivariable logistic regression analysis of predictors of cyst positivity in mice revealed that DAT positivity in mice, location of chicken (urban and periurban), midland altitude and age of chicken (≥13 months) were independent predictors of cyst positivity (Table 3).

**Table 3:** **Results of logistic regression analysis of predictors of** ***T. gondii*** **cyst positivity in mice**

**Variables **	**N** **(positive)**	**% (P)**	**Univariable**		**Multivariable**	
			**OR** **(95%** **CI)**	**P**-**value**	**OR** **(95%** **CI) **	**P**-**value**
Altitude						
Low land	30 (3)	10.0	1.0	-	1.0	
Mid land	124 (31)	25.0	3.0 (0.85, 10.58)	0.088	10.41 (1.21, 89.46)	0.033
High land	50(27)	54.0	10.57 (2.83, 39.40)	<0.001	1.51 (0.19, 12.10)	0.699
Breed						
Local	199 (58)	29.15	1.0			
Cross & exotic	5 (3)	60.0	3.65 (0.59, 22.40)	0.162	1.13 (0.11, 11.37)	0.919
Sex						
Male	40 (10)	25.0	1.0			
Female	164 (51)	31.1	1.35 (0.62, 2.98)	0.451		
Age						
≤ 6 months	34 (2)	5.88	1.0			
7-12 months	50(6)	12.0	2.18 (0.41, 11.52)	0.358	1.63 (0.23, 11.42)	0.621
≥ 13 months	120 (53)	44.17	12.66 (2.90, 55.23)	0.001	10.14 (1.97, 52.06)	0.006
Residence						
Rural	110 (25)	22.73	1.0			
U and Peri-U	94 (36)	38.30	2.11 (1.15, 3.88)	0.0116	3.78 (1.17, 12.17)	0.026
W2W1						
≤ 4.9 g	104 (30)	28.85	1.0			
≥5.0 g	100 (31)	31.0	1.11 (0.61, 2.02)	0.737		
DAT titer of chicken						
≤ 540	124 (33)	26.61	1.0			
≥1620	80 (28)	35.0	1.48 (0.81, 2.73)	0.203	1.52 (0.61, 3.81)	0.371
DAT status of mice						
Negative	104 (4)	3.85	1.0		1.0	
Positive	100 (57)	57.0	33.14 (11.3, 97.09)	<0.001	25.87 (8.05, 83.17)	<0.001

*DAT* = direct agglutination test, g = gram, N = tested number, P = prevalence, *OR* = odds ratio, U and Peri-U = (peri) urban, W2W1 = mean weight change of mice.

Sub-passage in mice or cell culture as well as cryopreservation of positive samples and permanent preparations were not done due to shortage of facilities. However, brain tissue homogenate of positive mice were kept deep frozen for future DNA extraction and genotyping elsewhere.

The high percentage of isolation of tissue cysts from seropositive chickens (59%, 24/41) is as expected since poultry production using extensive management of FR chickens is associated with *T. gondii* infection from the soil [2]. The present isolation rate was higher compared to 1 (2.3%) of 43 seropositive chickens from Addis Ababa, Ethiopia [5]. The variation in isolation rate between the present study and that of aforementioned study might be attributed to difference in the age of the chickens studied, number of chicken examined, the number of mice inoculated, the burden of *Toxoplasma* in chicken and the type and amount of tissues bioassayed [2,8,9]. In this study, we used five mice per sample to inoculate pooled brain and heart tissue homogenate of seropositive chickens which might have increased our success of isolation [9,10].

In the present study, *T. gondii* tissue cysts were detected from 61% (61/100) of seropositive mice while tissue cysts were observed in 2 DAT seronegative mice from 2 chickens. The logic behind the presence of cysts in the brain of seronegative mice could be due to low level of antibodies. Other possible reason can be due to the ability of *T. gondii* to escape immunity of host by avoiding fusion of lysosomes with phagocytes resulting in no or minimal antibody response [11]. The number of seropositive mice could have been higher if the cut-off value of DAT used was lower than 1:40 because viable *T. gondii* has been isolated from chicken with a DAT titer of 1:10 or even 1:5 elsewhere [9,12]. In contrary to the above, some seropositive mice (39%) had no cysts. This can be due to the small number and size of cysts in brain of mice which can be missed during counting of cysts under microscope [8]. Furthermore, the low sensitivity of microscopy, the low volume of brain examined under the microscope (30 μl), the absence of concentration method, and not performing further passaging of tissues of the mice without detectable cysts could have contributed for the absence of cysts from seropositive mice.

No clinical toxoplasmosis was observed in the mice during the monitoring period. This might suggest that most of the *T. gondii* strains circulating in the study area are not pathogenic to Swiss albino mice, *T. gondii* isolates in this study were not type I phenotypically or the dose of the parasite in the inoculated tissue homogenate of chicken was low. Similar finding has been reported from Addis Ababa, Ethiopia [5].

In accord with our findings, low cyst count was reported from FR chickens of Colombia, South America [13] and Ghana [10]. It has also been reported that poultry generally harbor less tissue cysts as compared to sheep, goats and pigs [14].

## Conclusions

*T. gondii* parasites are widespread in free range chickens of Central Ethiopia. This indicates that the environment is contaminated with *T. gondii* oocysts and that meat from free range chickens might be a source of infection for humans and other hosts. The local *T. gondii* isolates were not pathogenic for mice by bioassay. Altitude, DAT positivity of mice, age and urban and periurban location of chicken were independent predictors of cyst positivity in mice.
